# Very small embryonic-like stem cells have the potential to win the three-front war on tissue damage, cancer, and aging

**DOI:** 10.3389/fcell.2022.1061022

**Published:** 2023-01-04

**Authors:** Deepa Bhartiya, Nitu Jha, Anish Tripathi, Ashish Tripathi

**Affiliations:** Epigeneres Biotech Pvt Ltd, Mumbai, India

**Keywords:** stem cells, regeneration, cancer, iPS cells, VSELs

## Abstract

The concept of dedifferentiation and reprogramming of mature somatic cells holds much promise for the three-front “war” against tissue damage, cancer, and aging. It was hoped that reprogramming human somatic cells into the induced pluripotent state, along with the use of embryonic stem cells, would transform regenerative medicine. However, despite global efforts, clinical applications remain a distant dream, due to associated factors such as genomic instability, tumorigenicity, immunogenicity, and heterogeneity. Meanwhile, the expression of embryonic (pluripotent) markers in multiple cancers has baffled the scientific community, and it has been suggested that somatic cells dedifferentiate and “reprogram” into the pluripotent state *in vivo* to initiate cancer. It has also been suggested that aging can be reversed by partial reprogramming *in vivo*. However, better methods are needed; using vectors or Yamanaka factors *in vivo*, for example, is dangerous, and many potential anti-aging therapies carry the same risks as those using induced pluripotent cells, as described above. The present perspective examines the potential of endogenous, pluripotent very small embryonic-like stem cells (VSELs). These cells are naturally present in multiple tissues; they routinely replace diseased tissue and ensure regeneration to maintain life-long homeostasis, and they have the ability to differentiate into adult counterparts. Recent evidence suggests that cancers initiate due to the selective expansion of epigenetically altered VSELs and their blocked differentiation. Furthermore, VSEL numbers have been directly linked to lifespan in studies of long- and short-lived transgenic mice, and VSEL dysfunction has been found in the ovaries of aged mice. To conclude, a greater interest in VSELs, with their potential to address all three fronts of this war, could be the “light at the end of the tunnel.”

## Introduction

Over two decades ago, stem cells were thought to be “magic bullets” and were expected to completely revolutionize the field of medicine. However, despite huge investments, stem cells are not yet making significant contributions in the clinic (Bhartiya, 2019; Harvard Stem Cell Institute, 2022; Mallapaty, 2022). Despite the progress made in using human embryonic stem (hES) cells and induced pluripotent stem (iPS) cells, the use of hES cells continues to face challenges over ethical concerns; meanwhile, the existing roadblocks of tumorigenicity, immunogenicity, and heterogeneity prevent the clinical translation of hES/iPS cells. iPS cell lines show varying degrees of differentiation potential, associated genomic instability, and abnormal epigenetic status (Yamanaka, 2020).

Our team is especially intrigued by the recent attempts to convert iPS cells into mesenchymal stem/stromal cells (MSCs) (Eto et al., 2018; Xu et al., 2019; Liu, 2021; Rajasingh et al., 2021). Efforts like these make one wonder why iPS cells are used for paracrine support, rather than tapping into their regenerative potential. MSCs can be easily expanded from several sources, and even the microvesicles/exosomes derived from MSCs produce similar beneficial/regenerative effects upon transplantation (Zhang et al., 2020; Hade et al., 2021; Moghadasi et al., 2021). Does this mean that the future role of iPS cells will be reduced to providing exosomes? Several roadblocks still have to be overcome to bring iPS cells into the clinic.

### How MSCs fit into the landscape of regenerative medicine: The connection to very small embryonic-like stem cells

Unlike hES and iPS cells, MSCs have entered the clinics, and have shown abundant promise (Jovic et al., 2022). As we recently discussed (Bhartiya et al., 2022a), the beneficial effects of transplanting MSCs, as reported in multiple clinical trials (Squillaro et al., 2016; Esfandyari et al., 2020), come from their capacity to rejuvenate (i.e., improve the microenvironment/niche of) diseased tissues by providing paracrine support to the tissue-resident, endogenous very small embryonic-like stem cells (VSELs), which in turn regenerate the diseased tissues. There is an ongoing debate on the true nature of MSCs, particularly whether they are stem or stromal cells (Caplan, 2017). They provide a niche for the hematopoietic stem cells (HSCs) in the bone marrow (Kfoury and Scadden, 2015), MSCs are indeed not stem cells; they overlap with the body’s population of pericytes (Caplan, 2008). Similar beneficial effects are also obtained by transplanting MSC-derived exosomes (Zhao et al., 2020; Rezaie et al., 2022). MSCs have the characteristic property of being adherent in nature and show the potential to rapidly expand in culture. Similar to HSCs, which differentiate into multiple types of blood cells, MSCs differentiate into adipocytes, osteoblasts, myocytes, and chondrocytes; however, neither HSCs nor MSCs have regenerative potential, which implies they have no potential to transdifferentiate and regenerate diseased tissues of other tissue-type and lineages. HSCs, better defined as “lineage-restricted” and “tissue-committed” progenitors, obtained from the cord blood or the bone marrow have had a tremendous clinical effect on cell regeneration in blood-related diseases (Gluckman et al., 1989; Ratajczak et al., 2022). Earlier studies reported MSC differentiation into cell types of other lineages (ectoderm, mesoderm, and endoderm), but this can be explained by the presence of VSELs or multilineage-differentiating stress-enduring (MUSE) cells, which exist as a sub-population; MSCs themselves are not pluripotent (Bhartiya, 2013; Dezawa, 2016). MSCs provide a niche and the required paracrine support for tissue-resident stem cells to function and to undergo normal expansion/differentiation.

### An introduction to VSELs

**FIGURE 1:**
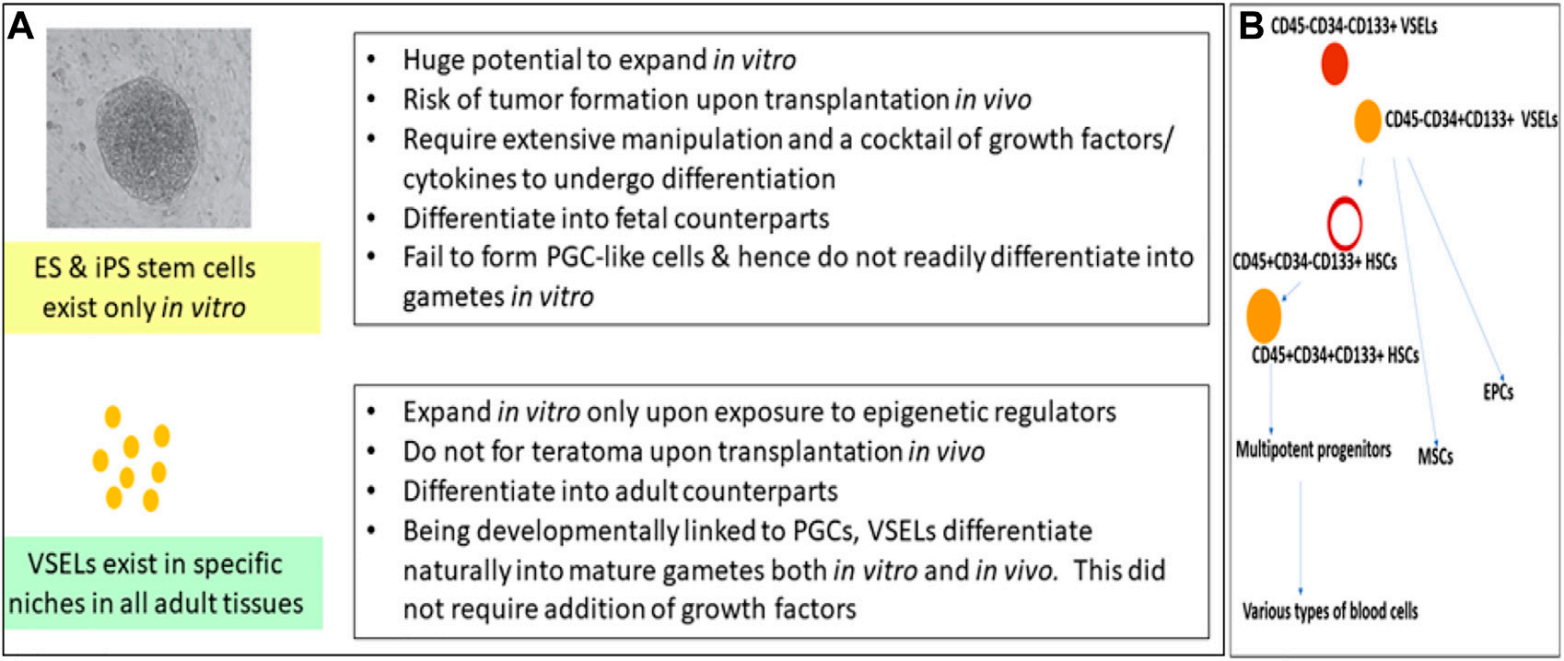
**(A)** compares VSELs with ES and iPS cells. Pluripotent VSELs exist in adult tissues; they regularly participate in adult tissue regeneration and remodeling, with no risk of teratoma formation. More focused research efforts are required to make use of their regenerative potential. **(B)** shows that VSELs sit at the top of the hematopoietic system hierarchy, and that they give rise to HSCs, MSCs, and EPCs. VSELs, very small embryonic-like stem cells; ES cells, embryonic stem cells; iPS cells, induced pluripotent stem cells; HSCs, hematopoietic stem cells; MSCs, mesenchymal stem cells; EPCs, endothelial progenitor cells.

VSELs were first reported in 2005 and were recently reviewed by Ratajczak et al. (2013) and Ratajczak et al. (2019). The germ-line origin of VSELs is the primordial germ cells (PGCs); being pluripotent, VSELs show the ability to differentiate into three germ layers, *viz.*, the ectoderm, endoderm, and mesoderm, and in gametes both *in vitro* and *in vivo*, in both mice and humans (Havens et al., 2014; Monti et al., 2017a; Lahlil et al., 2018; Shaikh et al., 2017; Ratajczak et al., 2019). They reside in all adult tissues and participate in regular regeneration and remodeling under physiological conditions (Figure 1A). They remain undifferentiated, maintain their distinct spherical shape, possess euchromatin, show dark-blue-stained nuclei upon hematoxylin–eosin staining, have a high nucleo-cytoplasmic ratio, and express pluripotent transcription factors with open chromatin at the OCT-4 promoter (Shin et al., 2009). VSELs have been studied for their global gene expression in mice (Shin et al., 2012) as well as in humans (Virant-Klun et al., 2013; Lahlil et al., 2018). The gene expression of VSELs has been summarized and compared with ES, iPS, and HSCs (Bhartiya et al., 2016). As with ES cells, the Ezh2-dependent bivalent domain mechanism contributes to the pluripotent state of VSELs (Shin et al., 2012). However, unlike ES and iPS cells, VSELs do not readily divide in culture, and upon transplantation *in vivo,* they neither form teratoma upon injection into immuno-deficient mice nor complement a growing embryo, two gold-standard tests for proving a pluripotent state. The inability of VSELs to achieve this state is basically because of their unique epigenetic status and their quiescent state due to the erasure of differently methylated regions (DMRs) at some of the paternally imprinted genes involved in embryogenesis (Shin et al., 2010; Ratajczak et al., 2019), which allow them to achieve what the hES/iPS cells cannot (Figure 1A). Recent attempts to expand VSELs *in vitro* by treating with epigenetic modulators, such as pyrimidoindole derivative (UM171) and nicotinamide acid, were successful (Lahlil et al., 2018; Domingues et al., 2022). CD34^+^ cells are now being produced on an industrial scale in a “good manufacturing practice” facility with an increased fraction of LIN^–^CD45^–^CD133^+^ VSELs (Henon et al., 2022). Meanwhile, Domingues et al. (2022) showed that CD34^+^ VSELs represent an excellent source of true vasculogenic endothelial progenitors for vascular repair, especially since both ES and iPS cells have failed to show similar potential. Ratajczak et al. (2011) differentiated VSELs into HSCs *in vitro* by culturing on OP9 feeder support. VSELs sit at the top of the hematopoietic system hierarchy and give rise to HSCs, MSCs, and EPCs (Figure 1B), which further differentiate into all types of blood cells, pericytes, and endothelial cells (Taichman et al., 2010; Domingues et al., 2022).

VSELs ensure replacement of tissue-committed progenitors throughout life and are activated to regenerate damaged organ tissue upon injury *in vivo*, e.g., in the lungs (Ciechanowicz et al., 2021), pancreas (Mohammad et al., 2020), bone (Leppik et al., 2020), and endometrial epithelium (Singh and Bhartiya, 2022). Being developmentally linked to PGCs, which are natural precursors for gametes, VSELs differentiate into oocytes and sperm *in vitro* (Bhartiya et al., 2017); however, it has proven difficult to differentiate human ES and iPS cells *in vitro* into PGC-like cells (PGCLCs), and thus, the hype surrounding the potential conversion hES and iPS cells into gametes for clinical use has not yet translated into reality. It is easy to regulate cellular differentiation by exposing stem cells to growth factors and cytokines *in vitro*, but modulating their epigenetic status is not yet possible. VSELs are also responsible for the regeneration of diseased organs in conditions where transplanted mesenchymal stromal cells provide paracrine support (Bhartiya et al., 2022a). Complete restoration of spermatogenesis from VSELs that survived chemotherapy was observed *in vivo* in busulfan-treated mice when MSCs were transplanted (Anand et al., 2016). VSELs and OSCs together are active in adult ovaries, resulting in oogenesis that is similar to spermatogenesis observed in males (Sharma and Bhartiya, 2021a). However, VSELs have remained elusive during the lineage-tracing studies reported so far because of their quiescent state; our recent work uncover their role in various biological processes *in vivo*, we have had success tracking fate of GFP-tagged VSELs (Bhartiya et al., 2022b).

**FIGURE 2:**
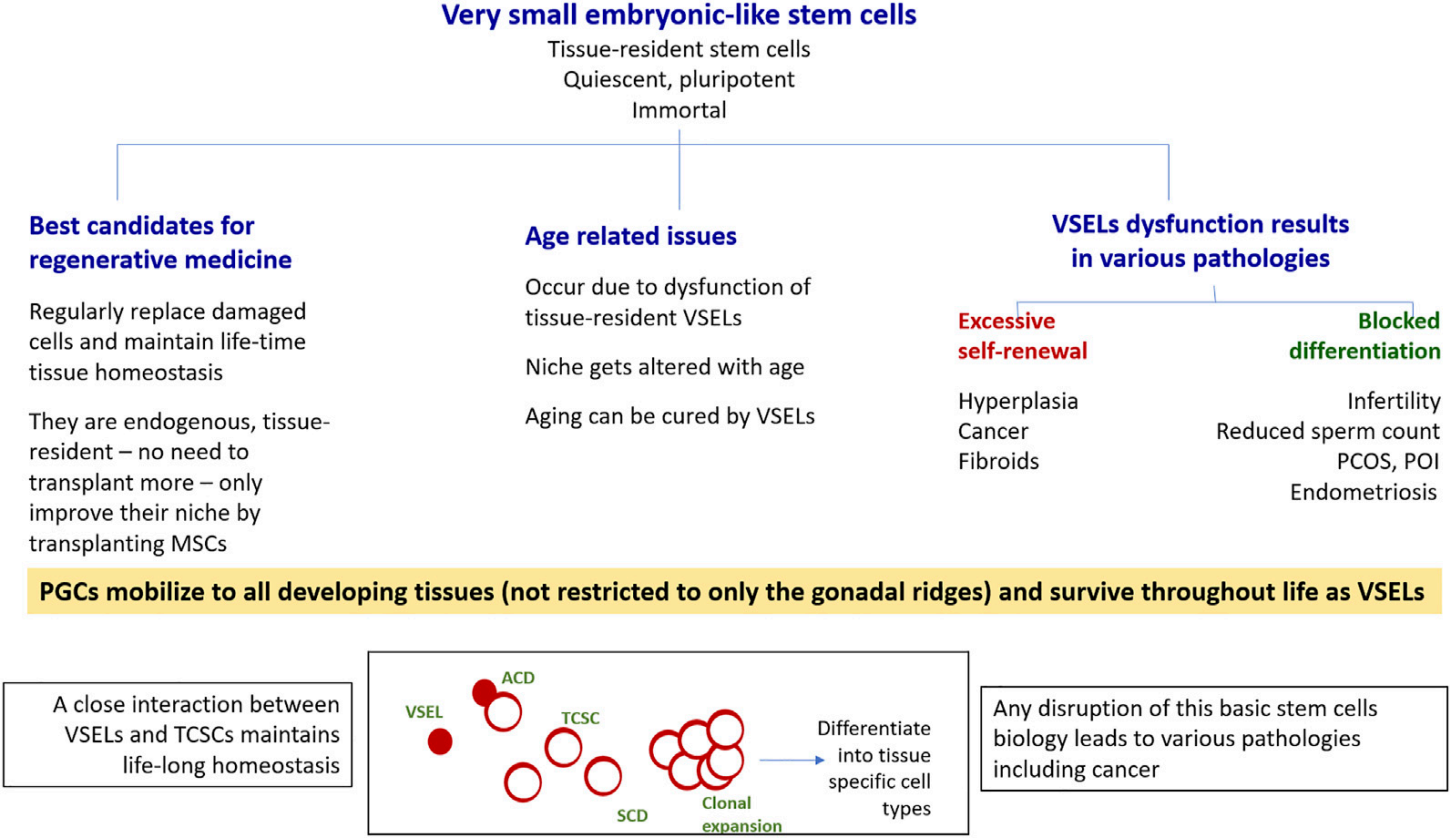
VSELs have the potential to win the war for human health on three major fronts. As shown, VSELs have the ability to regenerate adult tissues with none of the associated problems currently facing human embryonic and induced pluripotent stem cells, including ethical concerns as well as genomic instability, tumorigenicity, immunogenicity, and heterogeneity. Age-related disorders occur due to VSEL dysfunction. Various pathologies, including cancer, occur due to the dysfunction of the stem/progenitor cell compartment in adult tissues, as shown in the bottom panel. Excessive self-renewal of VSELs results in cancer, and the blocking of stem/progenitor cells differentiation (to different extents) gives rise to a wide spectrum of pathologies. This was first revealed through studying the effects of neonatal exposure to endocrine disruption on mouse reproductive tissues, but similar mechanisms could exist in other organs as well. More focused research on various aspects of VSELs is needed. VSELs, very small embryonic-like stem cells; PGCs, primordial germ cells; TCSCs, tissue-committed stem cells (progenitors); ACD, asymmetrical cell division; SCD, symmetrical cell division.

In addition to VSELs, adult tissues also harbor actively dividing and lineage-restricted tissue-committed stem cells (TCSCs) or the “progenitors” with limited plasticity, such as HSCs in the hematopoietic system and spermatogonial stem cells (SSCs) in the testes. VSELs serve as a back-up pool to give rise to tissue-committed progenitors and maintain life-long tissue homeostasis (Figure 2), and both VSELs and the tissue-specific progenitors are also mobilized when tissue function is affected. VSELs and progenitors express ERα, ERβ, and FSHR, and are thus vulnerable to various endocrine insults during early development. VSELs therefore have the potential to carry these insults into adult life, (since other somatic cells that also get exposed have limited life span and are regularly replaced by the VSELs), where these insults can manifest as a wide spectrum of pathologies, including cancer. It is the excessive self-renewal of pluripotent VSELs and their blocked differentiation (Figure 2) that initiate various diseases, including cancer (Bhartiya et al., 2022c).

### VSELs and HSCs: The CD34 connection

CD34 is a transmembrane phosphoglycoprotein, first identified on hematopoietic stem and progenitor cells. VSELs are at the top of the hierarchy that comprises HSCs, MSCs, and EPCs. VSELs are more primitive and give rise to progenitor HSCs (Taichman et al., 2010; Ratajczak et al., 2011a; Domingues et al., 2022) by undergoing asymmetrical cell divisions (Ganguly et al., 2018). VSELs are 2–6 µm cells with a surface phenotype of LIN^–^CD45^–^CD133^+^ in humans and LIN^–^CD45^–^SCA-1^+^ in mice, whereas HSCs have a surface phenotype of LIN^–^CD45^+^CD133^+^ in humans and LIN^–^CD45^+^SCA-1^+^ in mice. There is heterogeneity among VSELs and HSCs based on the expression of markers. Most intriguing is the expression of CD34 on the cell surface. Earlier, CD34^+^ HSCs had been thought to be the most primitive, but CD34^–^ HSCs have recently been reported, altering the stem cell hierarchy (Sonoda, 2021). This hierarchy of stem cells in the hematopoietic system needs further modification, as VSELs are the most primitive stem cells (Figure 1B).

We undertook a study on human cord blood and peripheral blood to evaluate the expression of CD34 on LIN^–^CD45^–^ VSELs and LIN^–^CD45^+^ HSCs via flow cytometry (Shaikh et al., 2017, unpublished data). The most primitive VSELs are LIN^–^CD45^–^CD34^–^, which exist along with LIN^–^CD45^−^CD34^+^ VSELs. A small fraction of LIN^–^CD45^+^ HSCs are CD34^–^, but the majority of them express CD34. CD34 is also expressed by MSCs, EPCs, and several other cells, but all these cells are also positive for lineage markers. Thus, as shown in Figure 1B, LIN^–^CD45^–^CD34^–^ CD133^+^ VSELs are the most primitive stem cells in the hematopoietic system, with the potential to regenerate all types of tissues due to their pluripotent state. On the other hand, HSCs possess limited potential in that they can only regenerate tissue damaged by blood diseases. The many failed efforts to regenerate other tissues using bone marrow- or cord blood-derived mononuclear cells taught the global scientific community that progenitors become lineage-committed and tissue-restricted, and have no potential to cross boundaries or transdifferentiate.

### VSELs and cancer

The somatic mutation theory (SMT) of cancer suggesting that mutations initiate cancer has recently been challenged by several groups in favor of the tissue organization field theory (TOFT), which describes cancer as “development gone wrong.” There have been calls for abandoning the SMT in favor of TOFT (Bizzarri and Cucina, 2016; Sonnenschein and Soto, 2020; Monti et al., 2022). The presence of embryonic markers in multiple cancers has intrigued the research community, and several researchers have postulated concepts such as the reprogramming and dedifferentiation of somatic cells to their pluripotent, OCT-4–expressing stem-like state to initiate cancer (Carvalho, 2020; Shivdasani et al., 2021). They postulate that a process similar to reprogramming somatic cells in a Petri dish to produce iPS cells may occur naturally in the body tissue, producing cancer stem cells and therefore leading to cancer initiation. Cancer is not a “genetic disease” but rather a stem cell disease; OCT-4–positive VSELs have been implicated in the initiation of cancers (rather than the random reprogramming of somatic cells) in multiple organs (Bhartiya et al., 2022c; Kaushik and Bhartiya 2022; Singh and Bhartiya 2022). Mutations may possibly occur as a consequence of the cancer, as the cells undergo rapid expansion.

We can make progress in the war against cancer by understanding the potential role of VSELs, as embryonic remnants, in initiating cancer (Ratajczak et al., 2009). Restoring the quiescence of VSELs and their normal functioning provides an interesting therapeutic target for the treatment of cancer.

### VSELs and aging

Aging is an inevitable part of life, and many different diseases, including cancers, Alzheimer’s disease, osteoporosis, cardiovascular diseases, and cognitive dysfunction, manifest as age increases. Interest in developing methods that can reverse the aging process, and thereby facilitate an extended period of life with rejuvenated organs, is growing; indeed, these efforts have attracted enormous amounts of attention and huge investments in recent years (Eisenstein, 2022). It has been demonstrated that short-term induction of Yamanaka factors has the potential to roll back cellular aging and repair tissues without reverting to pluripotency (Eisenstein, 2022). Partial reprogramming has reversed age-related phenotypes in eye, muscle, and other tissues in cultured mammalian cells, and even in genetically engineered rodent models, by countering epigenetic changes (Eisenstein, 2022). However, while these therapies have huge therapeutic potential to reverse age-related diseases, scientists are currently focused on more fundamental research. The exact mechanisms responsible for cellular reprogramming and how the epigenetic state becomes reversed remain to be understood. Ratajczak’s group has shown that the number of VSELs is correlated with longevity and caloric restriction, and that regular exercise and metformin have a positive effect on maintaining VSELs in adult tissues (Shin et al., 2010; Ratajczak et al., 2017). Laron and Ames dwarf mice with a deficiency of the GH receptor (GHR) were found to have severely reduced levels of plasma Igf1 and to live 30%–40% longer than their normal littermates; the number of VSELs in their bone marrow was 3-fold to 4-fold higher than normal during aging, However, both short-lived transgenic mice expressing bovine growth hormone (bGH) and wild-type animals injected with bGH for 2 months experienced an accelerated depletion of VSELs from the bone marrow, as well as premature aging (Ratajczak et al., 2011).

The ovary has been described as the pacemaker for physiological aging. Ovarian aging sets in earlier than aging in other organs of the body and, in turn, ushers in the start of various other diseases. Bhartiya’s group studied the potential role of VSELs in ovarian aging. VSELs reside in the surface epithelium of the ovary, along with the “progenitor” ovarian stem cells (OSCs) (Bhartiya and Patel, 2018). Under normal physiological conditions, VSELs and OSCs undergo neo-oogenesis and primordial follicle assembly in a regular manner across the estrous cycle (Sharma and Bhartiya, 2021b). In contrast, one study found that neonatal exposure to endocrine disruption in mouse pups (by treating them with estradiol and diethylstilbestrol) resulted in polycystic ovarian syndrome and primary ovarian insufficiency in adult life (Sharma and Bhartiya, 2022a). Epithelial cell smears from aged ovaries have shown VSELs, OSCs, and increased numbers of germ cell nests. Moreover, meiosis and further differentiation into oocytes were blocked. Thus, ovarian aging occurs due to VSEL dysfunction and a compromised niche (Sharma and Bhartiya, 2022b). The conversion of a pluripotent VSEL with open chromatin into OSCs involves extensive epigenetic changes, and this process is affected by aging. Ovarian aging can be reversed by transplanting MSCs directly into the ovaries (Tian et al., 2021; Wu et al., 2022) and by providing a younger niche. Aged ovaries possess rare premeiotic germ cells that can generate oocytes, following transplantation into a young host environment (Niikura et al., 2009). Tissue and organ rejuvenation and senescence/aging are closely related to the function of stem cells.

## Discussion

It has been more than 10 years since the publication of few studies that failed to detect VSELs via flow cytometry (Abbott 2013). But progress is the essence of life! The protocols to isolate VSELs have become more robust and easily replicable, and VSEL biology has matured over the last decade. VSELs have now been reported in multiple adult tissues by 60 independent groups (Ratajczak et al., 2019), and it is time to create a strong case for VSELs in the field and to resolve the question as to which are the “ideal” stem cell candidates for regenerative medicine. The resolution of the controversy surrounding VSELs is not an inconsequential “academic” pursuit; it directly impacts human health on three major fronts – i.e., regenerative medicine and the wars against cancer and aging (in terms of both diagnosis and treatment).

A failure to acknowledge the existence of VSELs could leave patients deprived of treatments on all three of these fronts. We therefore wish to inspire a new way of thinking, in the hope that it can spur rapid progress on these three fronts – especially in the war against cancer, which has been ongoing since 1971 – hence, for over five decades. Ignoring the presence of VSELs in adult tissues is no longer justifiable.

### VSELs and pluripotent stem cells: The similarities

• They express pluripotent markers.
• They demonstrate an ability to differentiate into three germ layers, as well as into germ cells (Kucia et al., 2006; Monti et al., 2017b; Shaikh et al., 2017).

### VSELs and pluripotent stem cells: The differences

• VSELs do not integrate into a developing embryo.
• VSELs do not form teratoma in SCID mice.
• VSELs do not divide and expand in culture; however, there has been some recent success in this regard (Ratajczak et al 2017; Lahlil et al 2018; Henon et al 2022).

### How VSELs compare to hES and iPS cells

• VSELs are tissue-resident, endogenous, pluripotent stem cells that are found in the body; hES and iPS cells exist only *in vitro*, as tissue-culture artefacts (Hasson et al., 2007), with no equivalent cells in the body.
• VSELs have unique features that keep them quiescent in nature; otherwise, cancers will spontaneously occur (Shin et al., 2009; Mierzejewska et al., 2013). The tumorigenic potential of pluripotent VSELs is therefore managed by natural body processes, whereas humans have not yet succeeded in resolving the tumorigenicity-related concerns associated with hES/iPS cells and/or their derivatives upon transplantation.
• VSELs, being developmentally linked to PGCs, differentiate into gametes *in vitro*—which has not yet been accomplished using hES/iPS cells (Virant-Klun 2018; Bhartiya et al., 2017 and 2014).
• VSELs differentiate into various adult cell types in the body *in vivo* and also functionally integrate—a feat that has not yet been achieved with transplanted iPS cells or their progenitors.
• Both human and mouse VSELs express steroid and gonadotropin hormone receptors; thus, they function under the influence of hormones within the body (Patel et al., 2013; Abdelbaset-Ismail et al., 2016; Bhartiya et al., 2021) and endocrine insults during early development lead to various pathologies due to VSELs dysfunctions.
• Unlike iPS and hES cells, which undergo symmetrical cell divisions in culture, VSELs possess a unique ability to undergo asymmetrical cell divisions, whereby they self-renew and give rise to a slightly bigger progenitor with a distinct fate (Bhartiya et al., 2018).

The average human life expectancy has increased in recent times, and the burden of age-related diseases has increased along with it. A developing field of research is focused on the reversal of the aging phenotype via *in vivo* partial reprogramming through introducing Yamanaka factors (Ocampo et al., 2016; Sikder et al., 2022). It is intriguing to see VSELs and Yamanaka factors at the crossroads of aging research, similar to making iPS cells. Many questions about cell reprogramming, both *in vitro* and *in vivo*, remain. In particular, it is unclear why only a very small proportion of somatic cells, not all of them, can be reprogrammed to their pluripotent state *in vitro*. It has been reported that a sub-population of pluripotent stem cells is the primary source of iPS cells in human fibroblast culture (Wakao et al., 2011). We postulate that introducing Yamanaka factors both *in vitro* and *in vivo* most likely stimulates VSELs, causing them to overcome quiescence and expand in the culture as iPS cells, allowing them to spontaneously reverse aging *in vivo*. However, better methods are needed: using vectors or Yamanaka factors *in vivo*, for example, is dangerous, and many potential anti-aging therapies carry the same risks as those using iPS cells. Similarly, organoids, which are currently attracting a lot of attention in the field of cancer biology, are formed on a regular basis due to clonal expansion and incomplete cytokinesis of tissue-resident VSELs and progenitors cells (Figure 1, Bhartiya et al., 2018).

To conclude, the scientific community seems to be lagging in the three-front war against tissue damage, aging, and cancer, especially where the use of stem cells to treat these conditions is concerned. The debate over which stem cells are “best” for regenerative medicine remains unsettled, and surprisingly, it is still not understood how cancer begins. It is our hope that greater interest in VSELs, and greater consideration of their potential role as a common answer to all three of these problems, will foster the creation of new methods to achieve regeneration, making it possible to both prevent cancer and treat it without causing recurrence. Moreover, aging is also a stem cell disease, and it too can be reversed by manipulating VSELs (Figure 1). A broader consensus in support of VSELs, based on published evidence, has the potential to lead to new ways of thinking that will help mankind.

## Data Availability

The original contributions presented in the study are included in the article/supplementary materials; further inquiries can be directed to the corresponding author.
